# Automatic segmentation of white matter hyperintensities in the elderly using FLAIR images at 3T

**DOI:** 10.1002/jmri.22004

**Published:** 2010-06

**Authors:** Erin Gibson, Fuqiang Gao, Sandra E Black, Nancy J Lobaugh

**Affiliations:** 1 Cognitive Neurology, Sunnybrook Health Sciences CentreToronto, Ontario, Canada; 2 Division of Neurology, Faculty of Medicine, University of TorontoToronto, Ontario, Canada

**Keywords:** white matter hyperintensity, FLAIR, seg-mentation

## Abstract

**Purpose:**

To determine the precision and accuracy of an automated method for segmenting white matter hyperintensities (WMH) on fast fluid-attenuated inversion-recovery (FLAIR) images in elderly brains at 3T.

**Materials and Methods:**

FLAIR images from 18 individuals (60–82 years, 9 females) with WMH burdens ranging from 1–80 cm^3^ were used. The protocol included the removal of clearly hyperintense voxels; two-class fuzzy C-means clustering (FCM); and thresholding to segment probable WMH. Two false-positive minimization (FPM) methods using white matter templates were tested. Precision was assessed by adding synthetic hyperintense voxels to brain slices. Accuracy was validated by comparing automatic and manual segmentations. Whole-brain, voxel-wise metrics of similarity, under- and overestimation were used to evaluate both precision and accuracy.

**Results:**

Precision was high, as the lowest accuracy in the synthetic datasets was 93%. Both FPM strategies successfully improved overall accuracy. Whole-brain accuracy for the FCM segmentation alone ranged from 45%–81%, which improved to 75%–85% using the FPM strategies.

**Conclusion:**

The method was accurate across the range of WMH burden typically seen in the elderly. Accuracy levels achieved or exceeded those of other approaches using multispectral and/or more sophisticated pattern recognition methods. J. Magn. Reson. Imaging 2010;31:1311–1322. © 2010 Wiley-Liss, Inc.

WHITE MATTER HYPERINTENSITIES (WMH) on T_2_-weighted images are common in many central nervous system disorders, including cerebrovascular disease and dementia. WMH are typically seen as diffuse signal increases in periventricular watershed regions, and as more focal “lesions” in deep white matter. Histologically, WMHs have been associated with a variety of pathological processes including edema, inflammation, demyelination, axonal loss, and gliosis (1–3), all of which result in increased T_2_ relaxation times. Although WMHs are often present in elderly persons who exhibit normal cognition, impaired cognition in both typical aging and dementia may be related to WMH burden (4,5). The possibility that WMHs have consequences for cognition in the elderly has motivated the development of segmentation methods to better assess this relationship.

Fast fluid-attenuated inversion-recovery (FLAIR) imaging offers advantages over conventional T_2_-weighted imaging for WMH detection because of the increased contrast between WMH and other brain tissues arising from the nulling of signal from cerebrospinal fluid (CSF). Several automated WMH detection methods have been proposed using FLAIR. Previous methods have been designed and validated at 1.5T using patients with arterial vascular disease (6), multiple sclerosis (7,8), Alzheimer's disease (9), and elderly patients (10–13). The majority of these WMH detection methods are multispectral, incorporating T_2_-weighted, and in some cases, proton density-weighted (PD), T_1_-weighted, and/or inversion recovery (IR) images together with the FLAIR image. Often, segmentation accuracy can be improved by increasing the separation between the different tissue types by incorporating higher-dimensional feature spaces (6) or support-vector machine approaches (13). However, the high contrast between WMH and healthy tissue on FLAIR images allows for the possibility of segmenting WMH using only FLAIR images. For example, Jack et al (9) developed a single-channel WMH segmentation method that used the histogram of the FLAIR image and a regression model to determine the appropriate intensity threshold for WMH, and more recently, Khayati et al (8) used a Bayesian classifier to segment MS lesions on FLAIR images at 1.5T.

One of the main challenges in developing an automated WMH segmentation using FLAIR is the minimization of false-positive classifications. False positives occur because the signal intensity in WMHs typically overlaps with that of normal tissue, even in the case of high-dimensional, multispectral segmentations. Two different approaches are commonly used to address this challenge. In one, false positives are minimized by identifying seed voxels with very high signal intensities on FLAIR or PD/T_2_-weighted ratio images (11,14). Segmentation is accomplished by using these seed voxels as input to a fuzzy-connectivity algorithm, which assesses the degree of fuzzy affinity between spatially connected elements. This approach successfully eliminates many false-positive classifications, especially those that occur at tissue interfaces. Unfortunately, this approach is often associated with increased false-negative classifications, such that there is a tendency to miss small focal WMH, as well as voxels well within larger WMHs. Residual false-positive classifications can also remain, particularly for artifacts resulting from CSF inflow (15). An alternative fuzzy-inference approach uses spatial priors from templates derived from segmented T_1_-weighted images (10). The primary errors with this approach are that hyperintensities outside of probable white matter are not included. However, the advantage of this approach is that false negatives in white matter are minimized.

The primary objectives of the present work were 1) to develop a robust automated method for segmenting WMH on FLAIR images at 3T, 2) to determine the precision of the method using synthetic data, and 3) to validate the accuracy of the method by comparing automatic and manual segmentations. A secondary objective was to determine the degree to which the automated results could be improved with a small amount of manual editing of the final segmentation results.

## MATERIALS AND METHODS

### Subjects

Images from 18 elderly individuals (nine males and nine females) with previously diagnosed white matter disease, and who were participating in research studies at Sunnybrook Health Sciences Centre, were included in this study. Participants were between the ages of 60 and 82 years (mean = 73.8, SD = 8.0). Informed consent was obtained in accordance with the Research Ethics Board.

### Magnetic Resonance Imaging (MRI)

FLAIR images were acquired at 3T (General Electric, Milwaukee, WI, software v. 12.4 and 12.5) using a quadrature head coil. Images were oriented parallel to the plane passing through the anterior and posterior commissure (2D T2FLAIR: TE = 140 msec, TR = 9300 msec, TI = 2200 msec, slice thickness = 3 mm matrix = 256 × 192, field of view [FOV] = 22 cm, phase FOV = 0.75). Two additional datasets, acquired in the same scanning session, were used for algorithm validation: a T_1_-weighted (3D, IR-prepped fast SPGR, TE ≊ 3.1 msec, TI = 300 msec, TR ≊ 7 msec, flip angle = 15°, slice thickness = 1.4 mm, NEX = 2); and a PD/T_2_-weighted sequence (dual-echo fast-spin echo, TEs = 20, 103 msec, TR = 2900 msec, echo-train length = 12, slice thickness = 3 mm, NEX = 2).

### Image Preprocessing

The WMH segmentation algorithm requires the removal of nonbrain tissues from the FLAIR image and the correction of intensity inhomogeneities. These two requirements can be achieved using any number of freely available software packages. We did not attempt to build these steps into the processing pipeline, especially as the efficacy of signal intensity corrections applied during image reconstruction are platform-dependent. For our FLAIR data the preprocessing requirements were achieved using N3 (16) (http://www.bic.mni.mcgill.ca/software/N3/; iterations = 150, stop threshold = 0.0001, distance = 55 mm) and FSL's bias correction procedures (17) (http://www.fmrib.ox.ac.uk/fsl/; “mfast” with default parameters) to correct intensity inhomogeneities, and FSL's Brain Extraction Tool (18) to generate a “brain mask” to remove nonbrain tissues.

The tests of the algorithm's precision required a segmented T_1_-weighted image (see below), so both manual and automatic WMH segmentations were performed on FLAIR images coregistered to the subject's T_1_-weighted image. For the segmentation protocol outlined below, the only required input was the masked, bias-corrected FLAIR image.

### Automated WMH Segmentation

**Figure 1:**
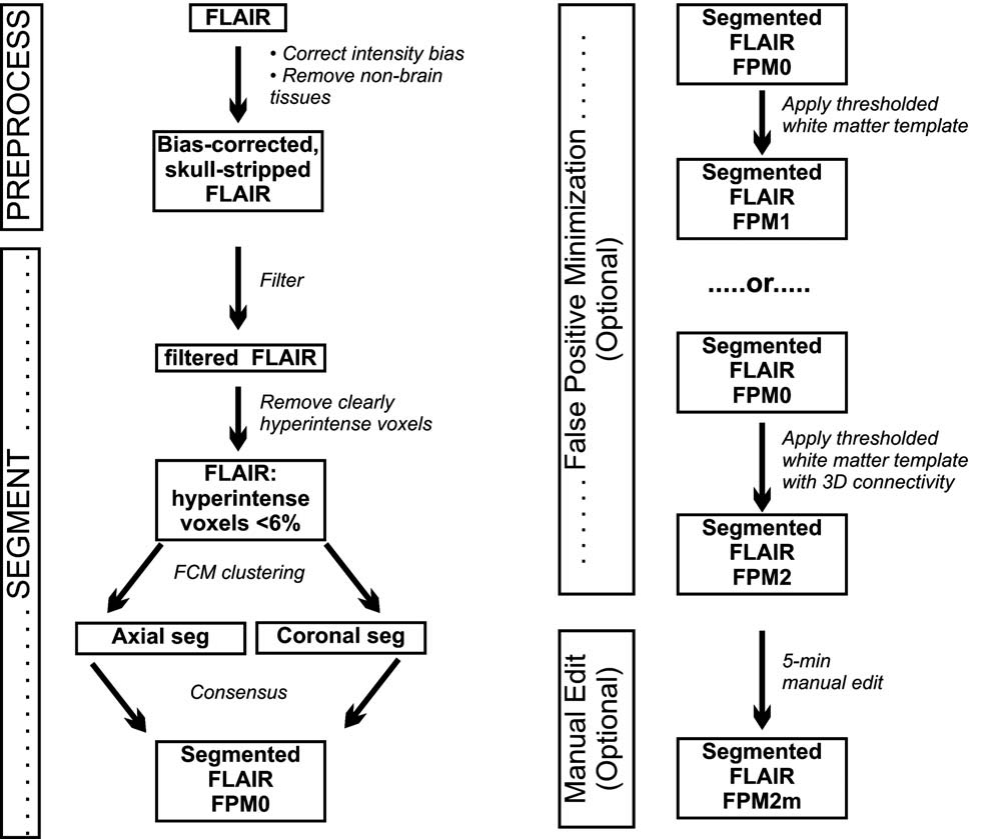
Flow diagram of the FLAIR FMH segmentation protocol.

The WMH segmentation protocol is fully automatic and has four steps: 1) noise-reduction filtering, 2) removal of clearly hyperintense voxels, 3) two-class fuzzy C-means clustering (FCM), and 4) thresholding to segment probable WMH. WMH segmentation is followed by an optional automatic false-positive minimization (FPM) step. The procedure requires ≈2.5 minutes on a Xeon E5440 processor with a clockspeed of 2.85 GHz. Full details are outlined in Fig. 1 and are described below.

#### Filtering

An edge-preserving anisotropic diffusion filter (19) (five iterations, time step = 0.0625, conduction = 1.95) was applied to the bias-corrected, skull-stripped FLAIR image. Only minimal smoothing was performed using the recommended time step and conduction parameters for 3D images (http://www.itk.org/ITKSoftwareGuide.pdf).

#### Removal of Unambiguously Hyperintense Voxels

This step was implemented to facilitate more accurate classifications at higher WMH loads. The FCM algorithm, in its standard form, requires that the datasets contain clusters that are roughly equivalent in size, which is the case for brain and background in most slices. Since these compartments both greatly outnumber hyperintense voxels, the two-class FCM generally works well. In our preliminary studies, when the slice WMH load was large (>≈6% of total brain slice volume), WMH volume was severely underestimated. It completely failed when the slice load was very large (ie, when WMH voxels outnumbered normal WM voxels). Removing unambiguously hyperintense voxels prior to applying the two-class FCM algorithm maintains the expected balance between brain, background-CSF, and hyperintense voxels on all slices.

**Figure 2:**
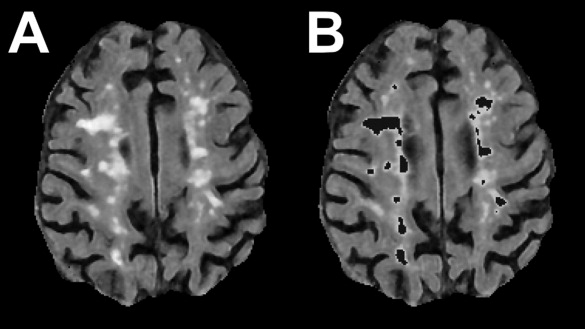
First-pass removal of voxels of obviously hyperintense voxels. **A:** Bias-corrected, skull stripped FLAIR image. **B:** Hyperintense voxels (signal intensity >4.25 on the intensity normalized FLAIR image) were removed from the original FLAIR image before applying the FCM algorithm.

The filtered FLAIR image was intensity-normalized to have a mean of zero and a variance of one. Examination of the normalized images from two subjects indicated that voxels with a normalized intensity greater than 4.25 were obviously hyperintense, regardless of slice location. This threshold was fixed, and all voxels above this threshold were removed automatically from the filtered FLAIR images, as shown in Fig. 2. To assess whether this manipulation reduced the size of the “hyperintensity” class to less than 6% of slice volume, the percent WMH load for each slice was calculated using manually traced WMH (see below), before and after removal of clearly hyperintense voxels.

#### Fuzzy Clustering

A two-class FCM algorithm was applied to the remaining voxels on a slice-by-slice basis. The FCM algorithm (20) is an unsupervised data clustering technique to partition datasets into “C” different clusters. Each data point is assigned a “fuzzy” membership grade that indicates the degree to which each data point belongs to each of the different clusters. It is a simple iterative procedure that works by minimizing an objective function representing the distance from each data point to the cluster means and weighted by the data point's membership grade. Voxels were assigned a membership grade in each of two classes: brain and background-CSF (range, 0.0–1.0). The FCM clustering algorithm was applied twice to each voxel, in the axial and coronal planes, and the consensus of the two segmentations defined the final segmentation. This processing step increased the robustness of the final segmentation results by removing voxels incorrectly classified as hyperintense on slices with small numbers of voxels.

#### Identification of Hyperintensities

**Figure 3:**
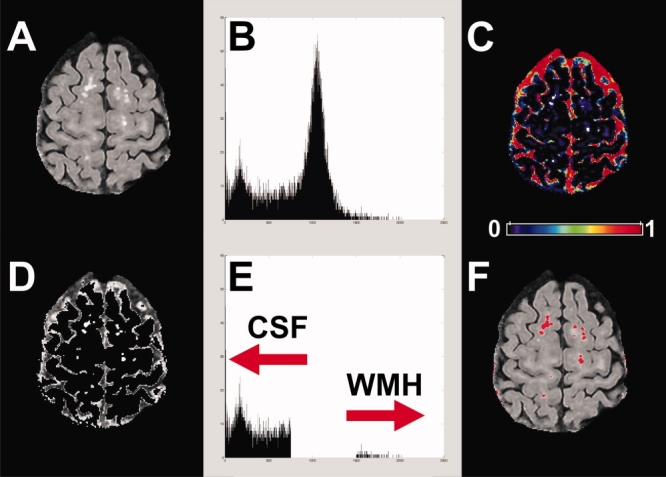
Segmentation of WMHs. **A:** Unmasked FLAIR image. **B:** FLAIR histogram. **C:** Pseudocolored image of FCM results for background-CSF class. Hyperintense voxels are assigned a membership grade greater than zero in this class. To isolate hyperintensities, a threshold was applied and the result was used to mask the FLAIR. Colorbar indicates membership grade. **D:** Masked FLAIR image. **E:** FLAIR histogram after removal of voxels below threshold. WMH voxels are now clearly separated from background-CSF voxels (arrows). For each slice, masked and unmasked histograms were compared, and the break in the histogram was used as the threshold for segmenting the WMHs. **F:** Final segmentation results overlaid onto the input FLAIR.

Hyperintense voxels are outliers in both classes and are assigned a membership grade greater than zero in each (Fig. 3C). The first step in the segmentation applies a threshold to the FCM results for the background-CSF class. The FCM results for two datasets with varying WMH load were used to define a membership grade above which voxels were visually hyperintense. The selected threshold was fixed and applied to all test datasets. The FLAIR data were masked, retaining only voxels with membership grades above threshold (Fig. 3D).

This masking step did not directly segment WMH, but did clearly separate background/CSF and WMH voxels based on FLAIR intensities (Fig. 3E). To obtain the final segmentation, two histograms were computed (bin width = 1 signal intensity unit). The first used all values in the filtered FLAIR image (Fig. 3B) and the second used only those voxels in the masked dataset (Fig. 3E). Starting from the right-hand tail of each histogram, the first bin found to contain unequal numbers of voxels was set as the intensity threshold for hyperintensities for the slice (right arrow, Fig. 3E). Only voxels classified as hyperintense in both axial and coronal planes were segmented as hyperintense (Fig. 3F).

### FPM

The algorithm as presented above produced acceptable results in many cases, but in all cases manual intervention (selecting and removal of false positives) would have improved the results. Our primary goal was to develop a fully automatic WMH segmentation that included minimal false positives. To remove the most common false positives, we used a white matter mask created from a thresholded probabilistic white matter template (Montreal Neurological Institute [MNI] “152,” available in the SPM software package; http://www.fil.ion.ucl.ac.uk/spm/) that had been registered to the FLAIR image. The template registration was accomplished by registering the MNI template T_1_-weighted image to the skull-stripped, bias-corrected, unfiltered FLAIR image (affine, FSL) and the resulting transformation matrix was used to move the white matter template into the subject's image space.

Two FPM strategies were tested. Both strategies involved thresholding the white matter template, and the threshold for each strategy was selected by examining the results of two subjects with varying degrees of WMH burden across slices. For the first (FPM1), the segmentation results were simply masked with the thresholded template (white matter probability = 0.41). For the second (FPM2), hyperintensities were removed if they were not connected in 3D to the thresholded template. As the 3D connectivity rule made FPM2 more liberal, a higher threshold was used (white matter probability = 0.63). Any hyperintense voxels remaining after the FPM step were classified as WMH. To assess the impact of the specific threshold values selected, six additional thresholds were tested, three above and three below the selected value, in increments of 0.04 white matter probability.

To determine whether limited manual editing could further improve the segmentation results, the automated WMH segmentation masks for FPM2 (WM probability threshold = 0.63) were subjected to a maximum of 5 minutes of manual editing per brain using the Analyze software package (Biomedical Imaging Resource, Mayo Foundation, Rochester, MN; http://www.mayo.edu/bir/Software/Analyze/Analyze.html). The following actions were allowed for the manual editing step: removal of false positives (eg, flow artifact) by relabeling them as background; adding back in portions of WMH truncated by the template by relabeling them as WMH class.

### Assessment of Segmentation Precision: Synthetic Data

The precision of the method was assessed by introducing synthetic “hyperintensities” into the image data. The synthetic data were created by adding known numbers of hyperintense voxels to brain slices that had no hyperintensities. Segmentation accuracy was assessed by comparing the results of the automatic protocol with manually traced WMH.

Images from six subjects with varying amounts of atrophy and low to moderate whole-brain WMH load were used. For each subject, three to four axial slices (mean, 3.4 ± 0.5) from the filtered FLAIR images were used. A total of 22 slices were selected across various levels of the brain, and the proportion of recovered voxels was used to index the accuracy of the algorithm. Details are described below.

#### Slice Preparation

A threshold for hyperintense voxels was identified manually for each selected slice, and any voxel exceeding this intensity threshold was removed. Slices were used only if less than 2% of the total slice volume was removed (mean percentage of removed voxels: 0.7% ± 0.5%).

#### Adding Synthetic Hyperintense Voxels

The manually identified WMH intensity threshold for each slice was used as the lower limit of synthetic data values. To find an appropriate upper limit, a histogram of the entire volume was calculated for each image (bin width = 1 signal intensity unit), and the value of the rightmost bin in the histogram containing four or more voxels was set as the upper limit. Thus, the lower limit was specific to each base slice, while the upper limit was calculated only once for each dataset. Synthetic data values were randomly selected from this range. This procedure ensured that artificial peaks were not created in the tail of the histogram and that no voxels were added with intensity values below what would reasonably be classified as a WMH. Note that because the segmentation operates strictly on the vector of FLAIR intensity values, it was not necessary to mimic the size or shape characteristics of typical WMH (eg, Ref. 21).

**Figure 4:**
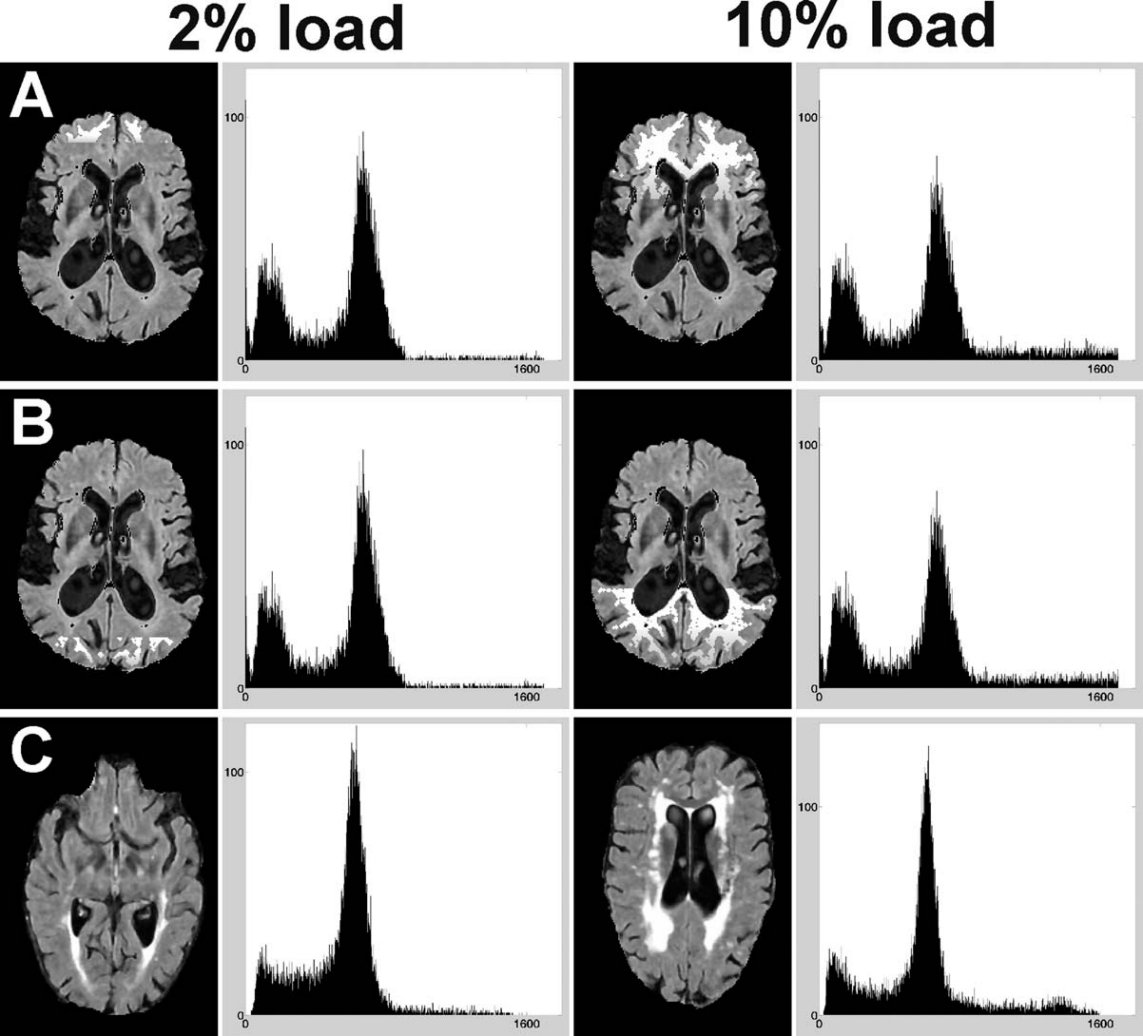
Synthetic and subject data at two levels of WMH load: 2% (left) and 10% (right) of total slice volume. Normal-appearing white matter voxels were identified from the subject's segmented T_1_-weighted image. Starting at the top **(A)** or bottom **(B)** of the slice, these voxels were replaced by synthetic data. Note that the signal intensities of the added voxels have been sorted to aid in visualization. **C:** Correspondence of histograms of subject data at load levels comparable to the synthetic datasets.

Finally, the subject's masked, bias-corrected T_1_-weighted image was segmented into gray matter, white matter, and CSF (22) to identify normal-appearing white matter (NAWM) voxels. A percentage of these voxels at the top or bottom of each slice was set to zero on the FLAIR images and replaced with the synthetic data. Two synthetic slices were created for each of 10 levels of WMH load (1%–10% of slice volume), generating 440 synthetic datasets. Figure 4A,B shows examples of locations to which WMH voxels were added. To aid in visualization, the voxel values have been sorted by signal intensity prior to filling. For comparison, images and histograms from representative slices from subjects with high and low WMH load are shown in Fig. 4C.

### Assessment of Segmentation Accuracy

An experienced neuroradiologist (F.G.), with specific expertise in tracing hyperintensities and stroke margins, manually segmented all WMHs on the bias-corrected FLAIR images using the Analyze software package. Image contrast was adjusted as needed for optimal determination of WMH extents. The outer boundaries of the WMH were defined initially using a threshold-based segmentation tool in Analyze and then the boundaries of each WMH were fully manually edited. Clearly hypointense necrotic or cystic regions within the hyperintensities were removed, as they were not included in the FLAIR segmentation. The subject's bias-corrected T_1_, PD, and T_2_-weighted images were used as additional sources of reference, but were not used explicitly to define the WMH margins. Based on the manually classified WMH volume, each subject was assigned to one of three levels of whole-brain WMH load: small (<10 cc, range 1.3–8.4 cc), medium (10–30 cc, range 12.0–25.2 cc), and large (>30 cc, range 30.4–80.3 cc).

### Algorithm Evaluation

The performance of the automated segmentation method was evaluated against the manual segmentation using four complementary similarity measures: the similarity index (SI), percent correct estimation (PCE), percent underestimation (PUE), and percent overestimation (POE), as used previously (6,7). The SI, defined as 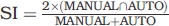, measures the spatial agreement between the automatic and manual segmentations, and takes into account both false-negative and false-positive classifications. The PCE, defined as 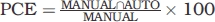, measures the percentage of correctly classified WMH voxels relative to the manual segmentation. The PUE, 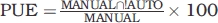, measures the percentage of missed voxels, and the POE, 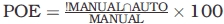, measures the percentage of false positives. For the synthetic data, the number of added voxels was used as the standard.

For the synthetic data, each similarity measure was analyzed using a repeated-measures analysis of variance (ANOVA, SPSS, Chicago, IL, v. 15.0.0, http://www.spss.com/) as a function of added hyperintensity load. The subject data were analyzed for one FPM threshold (see below) using a 3 (WMH burden: small, medium, large) × 3 (strategy: none, FPM1, FPM2) mixed-model ANOVA; and 3 (WMH burden) × 2 (strategy) ANOVAs were used to identify whether the FPM2 strategy with manual editing produced better results than FPM2 alone. Significant main effects and interactions were assessed using post-hoc Newman–Keuls analysis. Significance threshold was set at *P* < 0.05 for all analyses.

## RESULTS

### Removal of Clearly Hyperintense Voxels

**Figure 5:**
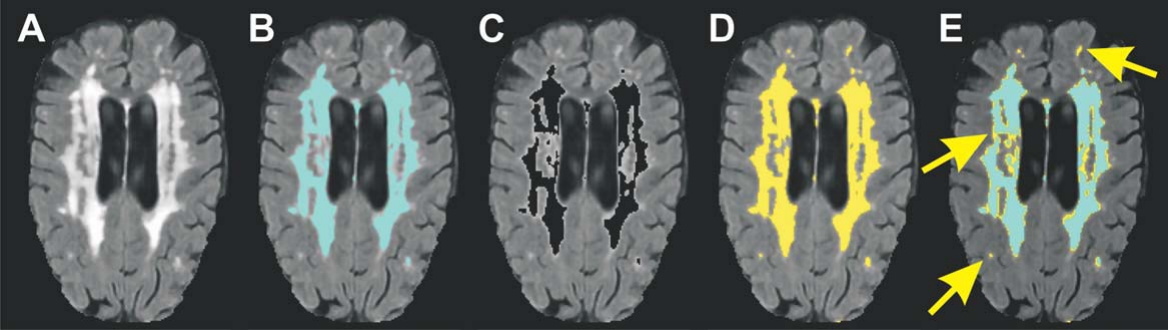
Effect of first-pass removal of clearly hyperintense voxels on WMH segmentation. **A:** FLAIR slice with high WMH load. **B:** Segmentation based on applying the FCM algorithm to the full dataset. **C:** Voxels above threshold on the intensity normalized FLAIR image are removed. **D:** FCM results for the masked dataset. **E:** True WMH volume can be underestimated when the two-class FCM algorithm is applied without removing clearly hyperintense voxels (yellow arrows).

WMH loads greater than 6% of the total slice volume were found on 8% of the manually traced slices. This was reduced to 0.8% of slices following the first-pass removal procedure, and the results were consistent across slice WMH load (detailed results are in tabular form in the online Supplemental Material). Note that the manual tracings do not include artifact voxels and therefore do not index the entire hyperintense volume for a given slice. However, because artifact voxels comprise only a small portion of the total slice volume (typically <≈1%), the results confirm that the two-class FCM requirement of two dominant tissue classes (brain and background-CSF) was met. If clearly hyperintense voxels were not removed, the segmentation underestimated the true WMH volume, as shown in Fig. 5. The first-pass removal procedure also improved segmentation accuracy: small WMH were more likely to be included, and the margins of larger WMH expanded and were more consistent with the manual tracing (arrows, Fig. 5E).

**Table 1:** Comparison of Two False-Positive Minimization Strategies

		Similarity measure^a^				
FPM strategy^b^ (*n*=16)	White matter template threshold (WM probability)	SI	Min SI	PCE	POE	PUE
0	—	0.65 ± 0.19	0.20	85.9 ± 7.9	117.9 ± 163.8	14.1 ± 7.9
1	0.29	0.80 ± 0.08	0.58	84.1± 8.5	27.8 ± 23.9	15.9 ± 8.5
1	0.33	0.80 ± 0.08	0.61	83.4 ± 8.7	24.7 ± 19.9	16.6 ± 8.7
1	0.37	0.81 ± 0.07	0.64	82.5 ± 9.0	22.4 ± 16.6	17.5 ± 9.0
**1**	**0.41**	**0.81 ± 0.07**	**0.67**	**81.4 ± 9.4**	**20.4 ± 14.3**	**18.6 ± 9.4**
1	0.45	0.80 ± 0.07	0.68	79.8 ± 9.7	18.8 ± 12.7	20.2 ± 9.7
1	0.49	0.80 ± 0.07	0.67	77.8 ± 10.3	17.4 ± 11.6	22.2 ± 10.3
1	0.53	0.78 ± 0.08	0.64	75.2 ± 11.2	16.0 ± 10.6	24.8 ± 11.2
2	0.51	0.80 ± 0.08	0.61	84.9 ± 8.2	29.2 ± 20.7	15.1 ± 8.2
2	0.55	0.81 ± 0.07	0.67	84.6 ± 8.2	25.6 ± 17.3	15.4 ± 8.2
2	0.59	0.81 ± 0.06	0.72	84.4 ± 8.2	23.6 ± 14.7	15.6 ± 8.2
**2**	**0.63**	**0.81 ± 0.06**	**0.70**	**83.8 ± 8.5**	**22.3 ± 13.6**	**16.2 ± 8.5**
2	0.67	0.81 ± 0.07	0.71	83.4 ± 9.2	21.7 ± 13.1	16.6 ± 9.2
2	0.71	0.82 ± 0.06	0.70	82.2 ± 9.7	19.2 ± 12.0	17.8 ± 9.7
2	0.75	0.81 ± 0.07	0.71	80.9 ± 10.5	17.7 ± 10.8	19.1 ± 10.5

a Compared to manual tracing. Values are mean ± standard deviation. SI: similarity index; Min SI: poorest performance; PCE: percent correct estimation; POE: percent overestimation; PUE: percent underestimation.

b FPM, false-positive minimization. 0: No minimization strategy. 1: Voxels below white matter template threshold were removed; 2: Voxels not connected in 3D to the thresholded white matter template were removed. White matter template thresholds used for additional testing are highlighted in bold.

### FCM Performance: Two-Plane Segmentations

Visually, only small differences were observed between the axial and coronal FCM results for most slices. False positives were frequently observed on small, superior slices in the axial plane. This occurred because an insufficient number of voxels were available for the two-class FCM algorithm. However, because voxels were required to be hyperintense in both planes, these misclassified voxels were eliminated from the final segmentation.

### FCM Performance: Synthetic Data

**Figure 6:**
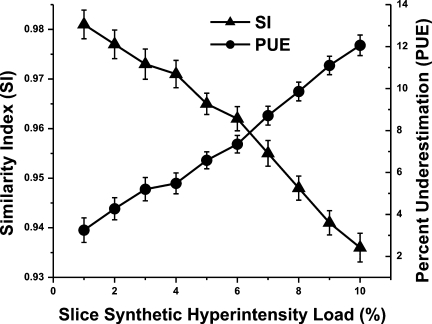
Changes in similarity index (SI) and percent underestimation (PUE) as a function of percentage of synthetic data added. Data are mean ± standard error of the mean.

**Figure 7:**
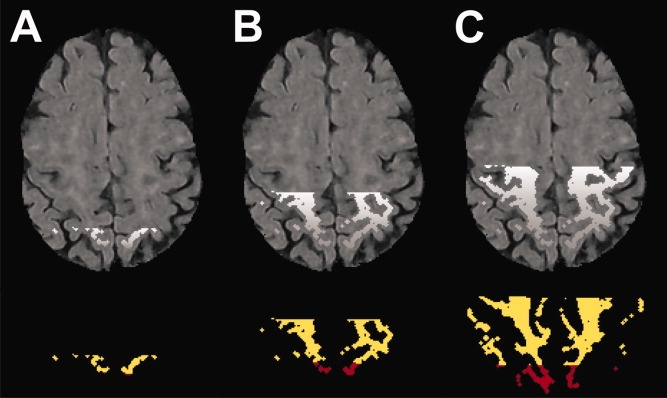
Segmentation of synthetic datasets. Top Row: Synthetic WMH voxels representing 1% (A), 5% (B), 10% (C) of total slice volume. Bottom Row: Yellow: correctly classified voxels; Red: missed voxels. For these slices, SI = 0.98, 0.95, 0.91; PCE = 96.5, 90.1, 83.9%; PUE = 3.5, 9.9, 6.1% for 1%, 5%, and 10%, respectively.

The results were consistent irrespective of slice location and where on the slice the synthetic data were added (top/bottom). Across the 10 levels of added hyperintense voxels, SI ranged from 0.98–0.93 (Fig. 6), with typical results shown in Fig. 7. Both SI and PCE decreased as the percent of synthetic data increased, while PUE increased (from 3.2% to 12.1%, Fig. 6, *F*(9,387) = 73.6 (SI); 86.8 (PUE)). Linear trends were significant for both measures (*F*(1,43) = 153.1 (SI); 167.2 (PUE)). Overestimations (POE) were rare (3% of slices; 1.67 ± 1.1%) and only at the two lowest levels of added voxels. Thus, the major bias in the procedure was to underestimate lesion volume, which increased slightly with WMH load.

### FPM: Improving Segmentation Accuracy

**Figure 8:**
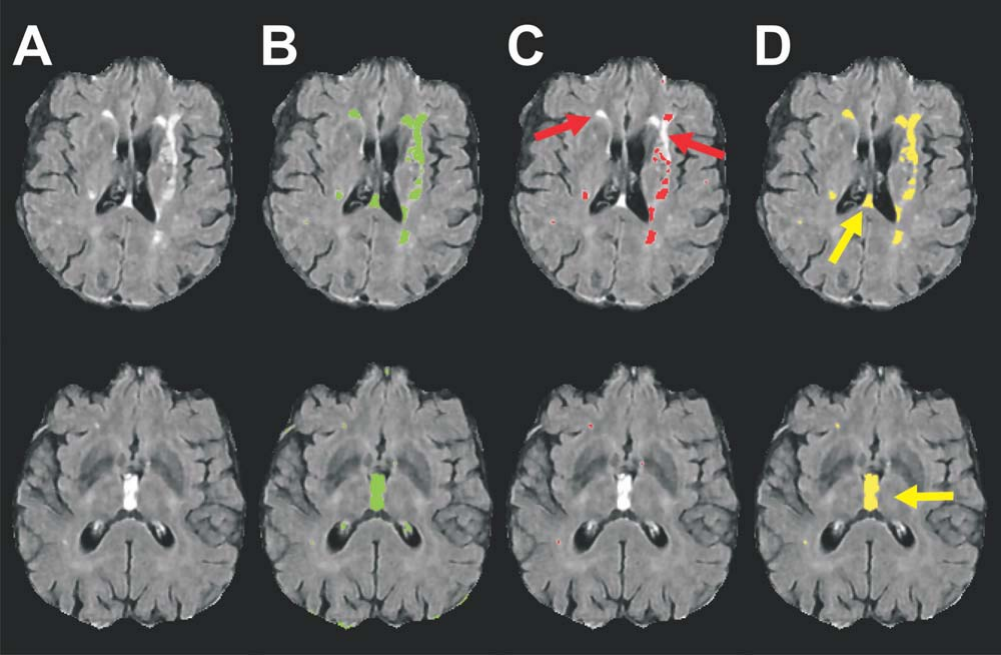
Errors associated with two FPM strategies. **A:** FLAIR image. **B:** Segmentation results with no FPM strategy. **C:** Segmentation results for FPM1. The primary source of error was false negatives (misses, red arrows). **D:** Segmentation results for FPM2. The primary source of error was false positives (yellow arrows).

**Figure 9:**
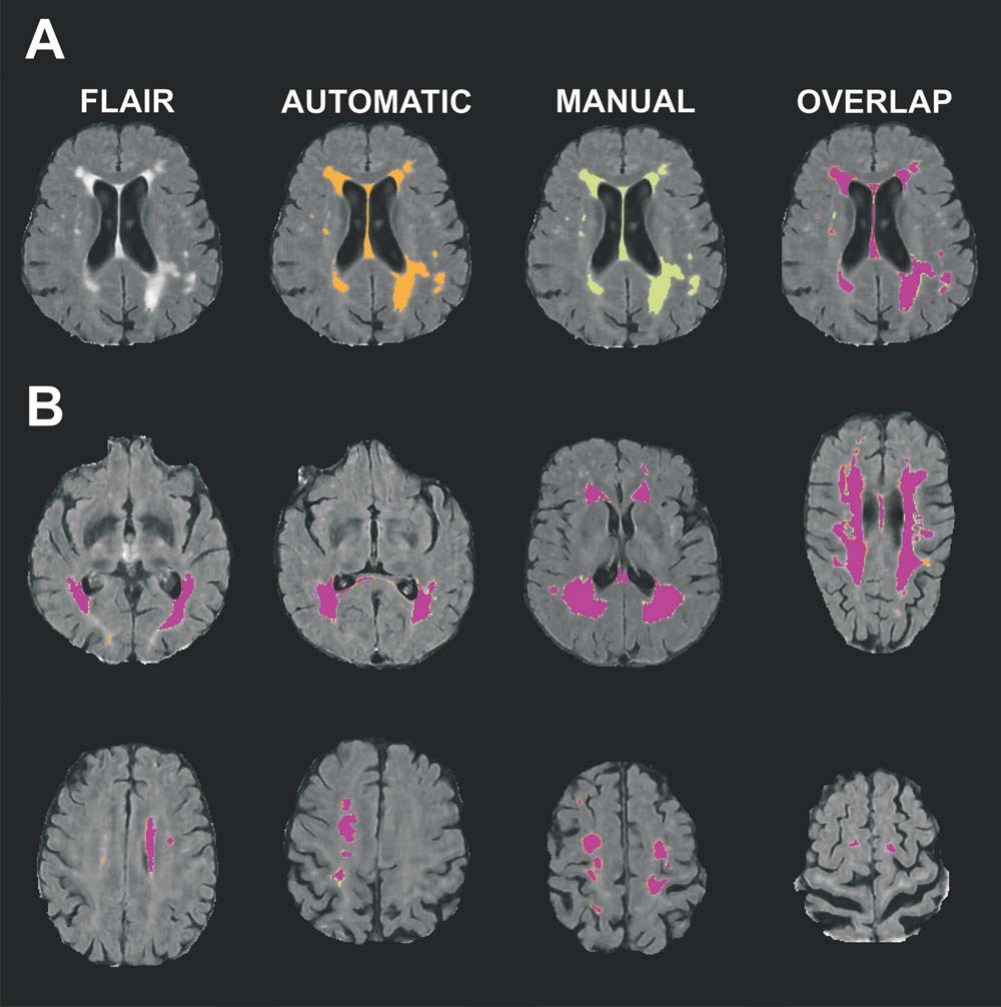
Comparison of automatic and manual segmentation results. False positives were minimized by accepting only hyperintensities connected in 3D to the thresholded white matter template (FPM2, white matter probability = 0.63). **A:** Comparison of automatic (orange) and manual tracing (green) results. Overlap of the two methods is shown on the far right. **B:** Overlap for slices representing varying degrees of WMH load and location. Magenta: voxels identified by both methods; Orange: automated method; Green: manual tracing.

The results of the two FPM strategies were assessed against the manual tracings. Across a range of template thresholds, both FPM1 and FPM2 substantially minimized the number of false positives (Table 1; Fig. 8), which resulted in much improved SIs for all template thresholds tested. Both FPM strategies showed a slight increase in the number of missed WMH voxels (PUE increased from 14% with no FPM to 16%–25% depending on threshold and strategy). No differences in SI were found between the two FPM strategies. The overlap of the FPM2 strategy with the manual tracing results across a range of WMH burden is shown in Fig. 9.

Using FPM1, FLAIR hyperintensities were routinely rejected in nonwhite matter structures, such as basal ganglia and thalamus. Additionally, portions of some WMHs were rejected when they extended beyond the thresholded white matter compartment. If the template threshold was lowered to incorporate the missed voxels, the improvement in underestimation was offset by a much larger number of false positives (cf. thresholds of 0.29 vs. 0.41 for FPM1, Table 1). The largest source of error in the FPM2 data was the inclusion of ventricular CSF flow artifact, which was seen in two of the 16 cases.

**Figure 10:**
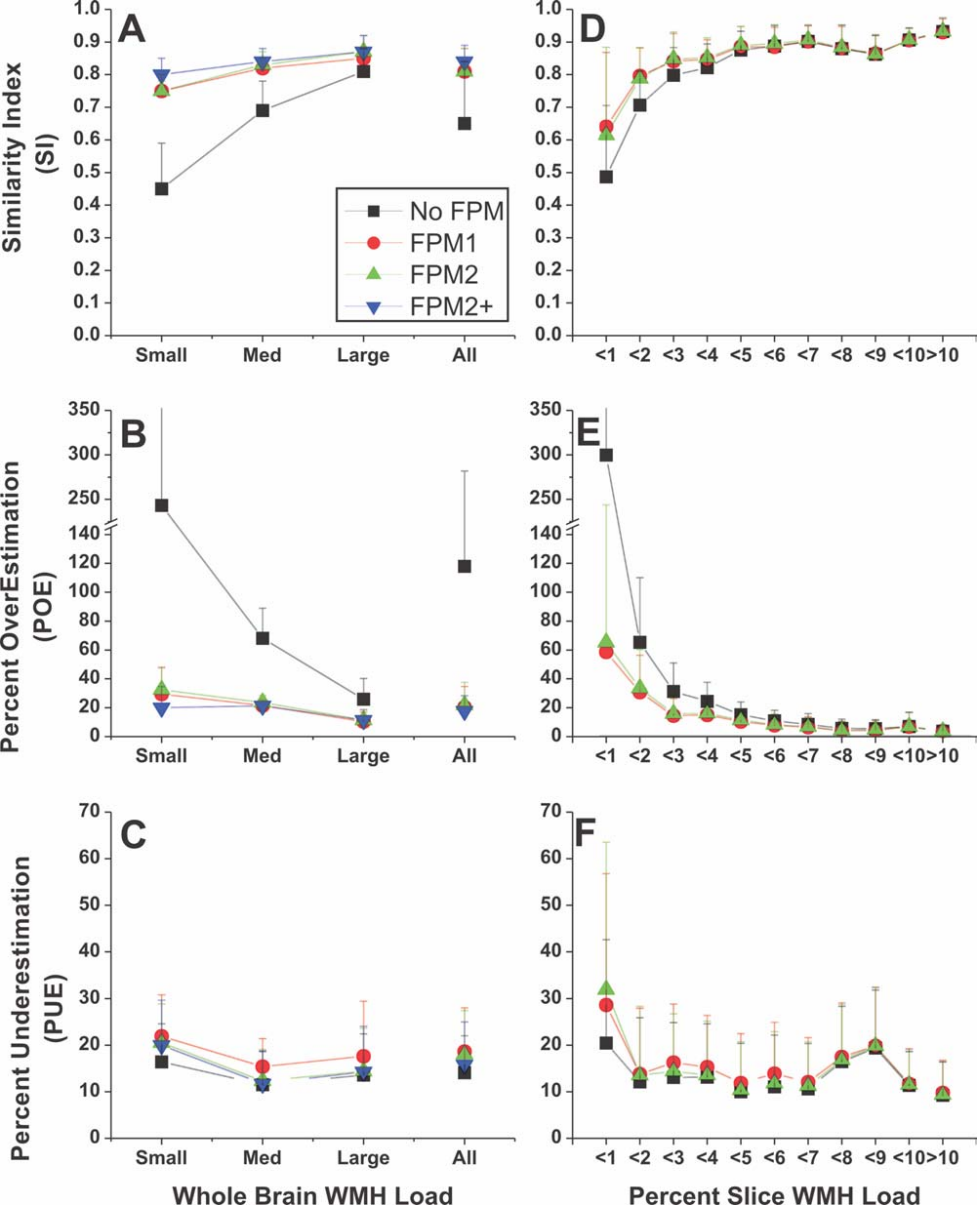
Similarity measures as a function of WMH burden and FPM strategy. Left panels: Whole-brain WMH load was designated as small (<10 cc), medium (10–30 cc), or large (>30 cc) based on manual tracing. Data are also shown collapsed across WMH burden: “All.” Improvements in SI **(A)** and POE **(B)** are seen with all FPM strategies, and were especially evident at small and medium WMH loads. Smaller improvements were seen for PUE **(C)**. Right panels. Similarity data are presented based on percent WMH found on the slice by manual tracing. As for the whole-brain results, the largest impact of the FPM methods on the SI **(D)** and POE **(E)** was at low WMH levels (<5%). PUE **(F)** was stable for load levels >2%. Black: No FPM, segmentation only; Red: FPM1, thresholded template applied to segmentation; Green: FPM2, thresholded template plus 3D connectivity constraint; Blue: FPM2+, FPM2 plus manual editing.

To further define the differences between the two FPM strategies, one threshold was selected for each method and the results were assessed as a function of WMH load. As whole-brain similarity measures are most commonly reported in the literature, we focused our assessment of the current method at this level (Fig. 10A–C; see online Supplemental Material for data in tabular form). Similarity measures at the slice level are also presented to compare with the simulated data (Fig. 10D–F).

Whole-brain similarity was significantly improved by both FPM measures for small and medium, but not large WMH burdens (SI, strategy × burden, *F*(4,26) = 12.3). The improved SI was largely due to the substantial reduction in overestimations. Overestimations were disproportionately large at small lesion loads with no FPM strategy (Fig. 10B; POE range: 77%–669%), and both FPM strategies successfully reduced POE in this case (POE range: 8%–53%). Assessment excluding the small WMH burden data showed that both FPM strategies successfully reduced POE at both medium and large WMH burdens (strategy × burden, *F*(2,16) = 8.5). The improvements in SI and POE were accompanied by small increases (≈4%) in underestimations across all burden levels (Fig. 10,C; *F*(2,26) = 15.8).

Manual editing was applied only to the FPM2 results, primarily because reclaiming the hyperintensities and partial hyperintensities missed by the FPM1 procedure would have required more than 5 minutes to accomplish. Most of the manual corrections to the FPM2 segmentations consisted of removing false positives. Manual editing was most beneficial when whole-brain WMH load was small, where POE was reduced from ≈32% to ≈20% (Fig. 10B; *F*(2,13) = 4.7).

At the level of individual slices, SI improved at load levels under 4%, and then stabilized at ≈0.90 for higher loads (Fig. 10D). Similar results were seen for overestimations, which reduced to ≈5% or less at higher loads (Fig. 10E). Underestimations stabilized more quickly to ≈10%, at WMH load levels >2% (Fig. 10F). The values at which each of these measures stabilized is comparable to the range of the precision of the method as assessed by the synthetic data at the highest load tested (Fig. 6).

## DISCUSSION

The primary aim of this work was to develop and validate a fully automatic method for segmenting white matter hyperintensities on 3T FLAIR images in the elderly. The current approach had excellent precision as assessed using synthetic data. It also fared well against manual tracing, a commonly-used “gold standard” for validating brain tissue segmentation algorithms.

The use of synthetic data provided an objective test of the algorithm's performance. The primary source of error derived from the tendency of the algorithm to underestimate the true hyperintensity volume. The magnitude of underestimation was linearly related to the number of added hyperintense voxels, and was restricted to voxels with the lowest intensities. Underestimation was also present in the subject data, and was typically at the margins of WMH, also regions of low signal intensity. However, in contrast to the results for the synthetic data, underestimation relative to the manually traced hyperintensities was most evident when slice WMH burden was less than 1% of slice voxels. In fact, both the similarity index and underestimation were quite stable across load levels, especially in the presence of the FPM strategies. This finding held when the total subject lesion load was used as the figure of merit. If clearly hyperintense voxels had not been removed from the data, our segmentations would have also failed on slices with the largest WMH loads. The consistency of the underestimation values across WMH levels speaks to the efficacy of the first-pass removal step in maintaining the correct balance among tissue classes. In addition, if the algorithm is in fact “missing” voxels at higher lesion loads in the subject data, they are also voxels that are not likely to be included by an experienced operator.

Specific features incorporated into the procedure to improve the accuracy of the method included the removal of clearly hyperintense voxels, the use of a consensus segmentation in two planes, and the incorporation of a white matter mask to define the probable spatial locations for WMH. The first-pass removal step was designed to ensure more robust and consistent operation of the FCM algorithm. Using the manually segmented data as a guide, 99% of slices had WMH loads ≤6% of the total slice volume after clearly hyperintense voxels were removed. Thus, the first-pass removal step effectively reduced the WMH load for each slice to a range optimal for the two-class FCM. In fact, the less-than-perfect performance at this step was restricted to eight slices from a single subject's data, where slice WMH load was between 7% and 11%. Although striking differences were not observed between the axial and coronal FCM results, the requirement that a voxel segment as hyperintense in both orientations avoided the need to reject image slices from analysis due to an insufficient number of voxels for the FCM algorithm.

Both automatic FPM strategies were successful across a range of template thresholds. Additionally, the results suggest that accurate WMH segmentations do not depend on precise registration, as a simple affine registration of the template was sufficient to reduce false positives. The two FPM methods differed statistically only with respect to under- and overestimations. For these metrics, the 3D connectivity rule (FPM2) outperformed the simple masking approach (FPM1). Given the small size of the differences (≈1%), the choice of which FPM strategy is optimal for a given dataset or application depends on whether or not manual intervention is deemed to be important to more completely reduce false positives for small WMH loads. If a fully automated approach is indicated, it may be more desirable use the thresholded white matter template directly as a mask (FPM1). While this strategy was insensitive to WMH located outside the boundaries of typical white matter, and thus produced the largest PUE, it was not as likely to include CSF flow artifact. On the other hand, simply excluding hyperintensities not connected in 3D to the white matter template (FPM2) brought underestimations to their best level, and adopting a semiautomatic strategy further improved both SI (≈2%) and POE (≈5%). The semiautomated procedure using FPM2 required the least amount of manual reclassification, because there was a greater initial agreement with the manual tracings, and there were no instances where subsections of WMH were misclassified.

The primary sources of error with this segmentation procedure are directly linked to the sensitivity of the FLAIR sequence. CSF inflow artifact can be a major source of error on 2D FLAIR imaging (≈50%) (15), but was not prominent in the current dataset (≈10%). However, prominent CSF flow artifact was easily eliminated as a major source of false positives by both FPM methods. Second, there is continuing debate in the literature regarding the relevance of thalamic infarcts, lacunes, and cystic white matter hyperintensities (23–25) for cognitive decline and progression of dementia. If these “black holes” on T_1_-weighted images are in fact CSF-filled, they will tend to be black holes on FLAIR images, and will not be included in the WMH burden volume (eg, 26). Incorporating those hypointense voxels as a unique tissue compartment would require separate segmentation of PD and/or T_1_-weighted images. One of our ultimate goals is to combine WMH and T_1_ segmentation results to achieve regional lobar tissue volumes (27). Therefore, the WMH segmentations and tracings were performed on FLAIR images coregistered to T_1_-weighted images. Performing the analysis in native acquisition space would avoid introducing interpolation error, and may be an important factor to consider in certain instances. However, the high similarity indices achieved indicate the procedure is robust with respect to the error introduced from interpolating the image into the higher resolution space.

The whole-brain accuracy of the present approach equaled or exceeded those derived from methods optimized for both single- and multichannel data at 1.5T. Our approach exceeded that of Admiraal-Behloul et al (10), who found SIs ranging from 0.70–0.82 across three similar levels of WMH load using a multispectral, fuzzy segmentation. At large lesion loads, our method had SIs similar to those derived using K-nearest neighbor (6) or multispectral fuzzy connectivity approaches (7). At smaller whole-brain WMH loads, the current approach appears to have outperformed both. At the slice level, high similarity indices and low underestimations were achieved consistently at slice loads >3% when an FPM strategy was used, and at slice loads >4% when the basic segmentation was used.

An additional advantage of this approach is that it is straightforward to implement. Neither large training datasets or labor-intensive manual labeling of hyperintensities are required. For a given set of FLAIR acquisition parameters, only two thresholds need to be established. The first threshold is derived from normalized images and is used to exclude obviously hyperintense voxels, so that slice WMH load is roughly 6% in the most severely affected cases. This threshold can be determined on a representative dataset with moderate to high WMH load. The second threshold establishes which voxels will be considered outliers in the background/CSF class. Very high similarity indices in the test dataset were achieved using only two subjects to establish the optimal values for both thresholds. Because the segmentation procedure has been designed and validated to perform equally well across a wide range of lesion loads, appropriate segmentation parameters can be selected by visual reference to only a small sample of test images.

In conclusion, the method presented here is the first, to our knowledge, to optimize segmentation of WMH in the elderly on 3T FLAIR images. This unsupervised method does not require extensive training sets or manual segmentations. It is based on a quick and simple clustering algorithm which, in conjunction with a template-based FPM strategy, meets or exceeds the results from more complex multichannel and/or pattern recognition techniques. Importantly, it delivers consistent results across a range of WMH loads, suggesting it will be useful in larger cohort studies of WMH in aging and dementia.
