# Supplementary material for: Automatic segmentation of white matter hyperintensities in the elderly using FLAIR images at 3T
Source: J Magn Reson Imaging. 2010 Jun;31(6):1311–22. doi: 10.1002/jmri.22004 (PMC2905619; doi:10.1002/jmri.22004)
Supplement: Supplementary file 1 [file jmri0031-1311-SD1.doc]

**Supplementary Table**

Similarity measures for three levels of whole-brain WMH load.

|  | | |  | Similarity Measure* | | | | |
| --- | --- | --- | --- | --- | --- | --- | --- | --- |
| WMH  Load | | | N | SI | PCE | | POE | PUE |
| No FPM** | | | | | | | | |
|  | | Small | 6 | 0.45 ± 0.14 | 83.6 ± 8.2 | | 243.2 ± 221.5 | 16.4 ± 8.2 |
|  | | Medium | 4 | 0.69 ± 0.09 | 88.5 ± 7.1 | | 68.1 ± 20.8 | 11.5 ± 7.1 |
|  | | Large | 6 | 0.81 ± 0.07 | 86.4 ± 8.8 | | 25.9 ± 14.3 | 13.6 ± 8.8 |
|  | | All Subjects | 16 | 0.65 ± 0.19 | 85.9 ± 7.9 | | 117.9 ± 163.8 | 14.1 ± 7.9 |
| FPM1** | | | | | | | | |
|  | | Small | 6 | 0.75 ± 0.05^†^ | 78.1 ± 8.9 | | 29.5 ± 18.5^†^ | 21.9 ± 8.9 |
|  | | Medium | 4 | 0.82 ± 0.03^†^ | 84.6 ± 6.0 | | 21.5 ± 3.8^†^ | 15.4 ± 6.0 |
|  | | Large | 6 | 0.85 ± 0.07 | 82.4 ± 11.8 | | 10.5 ± 6.5 | 17.6 ± 11.8 |
|  | | All Subjects | 16 | 0.81 ± 0.07^†^ | 81.4 ± 9.4^‡^,^‡‡^ | | 20.4 ± 14.3^†^ | 18.6 ± 9.4^‡^,^‡‡^ |
| FPM2** | | | | | | | | |
|  | | Small | 6 | 0.75 ± 0.03^†^ | 79.5 ± 8.3 | | 32.4 ± 15.1^†^ | 20.5 ± 8.3 |
|  | | Medium | 4 | 0.83 ± 0.04^†^ | 87.7 ± 6.8 | | 23.6 ± 4.9^†^ | 12.3 ± 6.8 |
|  | | Large | 6 | 0.87 ± 0.05 | 85.6 ± 9.2 | | 11.4 ± 6.8^†^ | 14.4 ± 9.2 |
|  | | All Subjects | 16 | 0.81 ± 0.07^†^ | 82.0 ± 9.4^‡^ | | 21.9 ± 15.7^†^ | 18.0 ± 9.4^‡^ |
| FPM2 Plus Manual Editing | | | | | | | | |
|  | Small | | 6 | 0.80 ± 0.05 | | 84.2 ± 9.2 | 20.0 ± 14.7^††^ | 20.0 ± 9.6 |
|  | Medium | | 4 | 0.84 ± 0.04 | | 88.3 ± 7.1 | 21.4 ± 3.2 | 11.7 ± 7.1 |
|  | Large | | 6 | 0.87 ± 0.05 | | 85.7 ± 9.7 | 11.4 ± 7.3 | 14.3 ± 9.7 |
|  | All Subjects | | 16 | 0.84 ± 0.05^††^ | | 84.2 ± 9.2 | 17.1 ± 10.6^††^ | 15.8 ± 9.2 |
|  | | | | | | | | |

* Compared to manual tracing. SI: Similarity Index; PCE: Percent Correct Estimation; POE: Percent Over Estimation; PUE: Percent Under Estimation. Values are mean ± standard deviation.

** FPM: False Positive Minimization. No FPM: No FPM strategy was applied to WMH segmentation. FPM1: Voxels below white matter template threshold were removed, WM Probability = 0.41; FPM2: Voxels not connected in 3D to the thresholded white matter template were removed, WM Probability=0.63.

^†^ Significant improvement over no FPM; ^††^Significant improvement over FPM2 (all *p*s < 0.05).

^‡^ Significantly poorer than no FPM. ^‡‡^Significantly poorer than FPM2.
